# The enigmatic helicase DHX9 and its association with the hallmarks of cancer

**DOI:** 10.2144/fsoa-2020-0140

**Published:** 2020-11-02

**Authors:** Chloe Gulliver, Ralf Hoffmann, George S Baillie

**Affiliations:** 1 Institute of Cardiovascular & Medical Science, College of Medical, Veterinary & Life Sciences, University of Glasgow, Glasgow, UK; 2 Philips Research Europe, High Tech Campus, Eindhoven, The Netherlands

**Keywords:** biomarker, cancer, DHX9, helicase, medicine, target, tumor

## Abstract

Much interest has been expended lately in characterizing the association between DExH-Box helicase 9 (DHX9) dysregulation and malignant development, however, the enigmatic nature of DHX9 has caused conflict as to whether it regularly functions as an oncogene or tumor suppressor. The impact of DHX9 on malignancy appears to be cell-type specific, dependent upon the availability of binding partners and activation of inter-connected signaling pathways. Realization of DHX9’s pivotal role in the development of several hallmarks of cancer has boosted the enzyme's potential as a cancer biomarker and therapeutic target, opening up novel avenues for exploring DHX9 in precision medicine applications. Our review discusses the ascribed functions of DHX9 in cancer, explores its enigmatic nature and potential as an antineoplastic target.

## Lay abstract

DExH-Box helicase 9 (DHX9) is an enzyme with multiple important functions in cells, therefore, its deregulated activity can cause alterations in cellular growth and subsequent formation of tumors. In particular, irregular DHX9 activity is attributed to the development of several hallmarks of cancer, however, it can have both pro- and anti-cancer effects, thus leading to conflicting views on DHX9's role in cancer development. The diverse implications of DHX9 in malignancy exposes the protein as a potential marker for cancer detection and intervention. Our review discusses the vast array of DHX9’s functions in cancer development, and its potential as a target for cancer treatment.

Helicase proteins have a diverse range of cellular functions that stem from their ability to resolve and remodel DNA and RNA structures. DExH-Box helicase 9 (DHX9), also known as RNA helicase A (RHA), belongs to the DExD/H-Box superfamily II of helicases [1]. The profile of DHX9 has rapidly increased in the oncology field lately because of two main factors. Firstly, the enzyme has been implicated in the control of many cellular processes which are often deregulated in cancer, such as genomic stability, transcription and DNA replication [2,3]. Secondly, DHX9 is overexpressed in many tumors including lung and colorectal cancers and for this reason, it has recently been proposed as a potential therapeutic target [4–6]. This review focuses on the role of DHX9 in the development and progression of cancer, highlights DHX9 as a potential biomarker for the evaluation of cancer progression and discusses strategies for targeting DHX9 as novel therapeutic avenues for cancer therapy.

## DExD/H-box RNA helicases

The unwinding and remodeling of DNA/RNA structures is ATP dependent and involves the catalytic action of helicases [7]. DExD/H-box RNA helicases are categorized as members of the helicase superfamily II by virtue of their conserved motif DExD/H amino acid signature sequence, which represents the core domain at which NTP binding and hydrolysis occur. While structurally similar, the DExD and DExH families contain slight diversity within the conserved DExD/H motifs [8]. Another difference between the families is that DExH helicases are able to hydrolyze all NTPs, while DExD helicases are specifically ATP driven [9]. It should be noted that although both types are best known for their ability to unwind RNA, they also have important roles in RNA metabolism via remodeling ribonucleoprotein complexes, processing pre-mRNA and catalyzing ribosomal RNA biogenesis [10,11]. These functions are essential in the regulation of transcription and translation, and coordination of gene expression [1,12].

## DHX9 general features & functions

**Figure 1.**
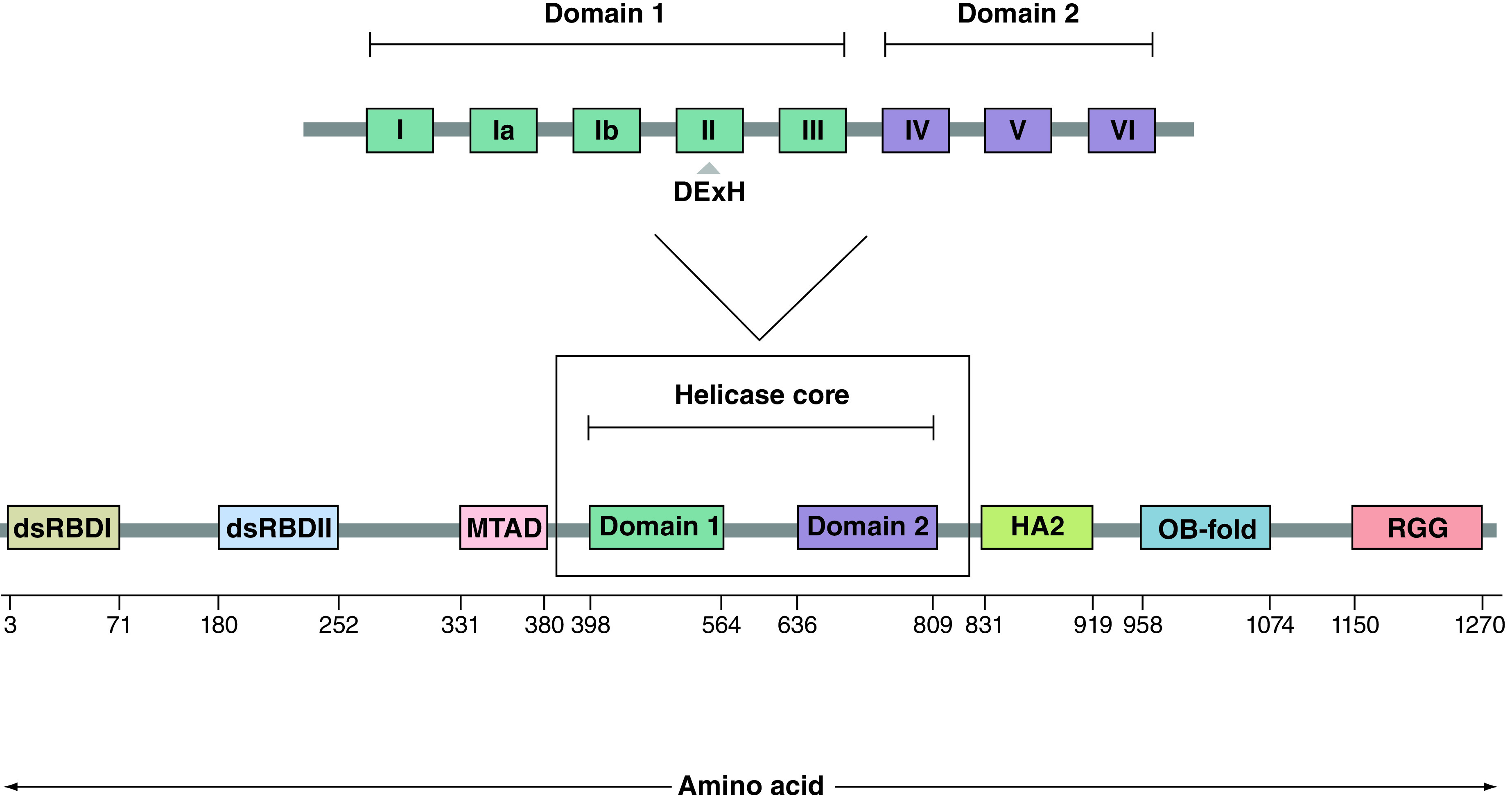
Human DExH-Box helicase 9 protein structure, domains and motifs. The entire protein is 1280 amino acids in length, with amino acid positions of domains and motifs shown as determined by UNIPROT. The helicase core domain consists of two distinct regions – the helicase ATP-binding domain (domain 1) in which the DExH motif is located, and the helicase C-terminal (domain 2). dsRBD I/II: Double-stranded RNA-binding domain I/II; Domain 1: Helicase ATP-binding domain; Domain 2: Helicase C-terminal domain; HA2: Helicase-associated domain 2; MTAD: Minimal transactivation domain; OB-fold: Oligonucleotide- or oligosaccharide-binding fold; RGG: Repeated arginine and glycine-glycine.

The *DHX9* gene, a DExH-box helicase family member, maps to chromosome 1q25 in humans, a location that has been identified as the major prostate cancer (PCa) susceptibility locus [13]. The encoded protein has a molecular weight of 140 kDa and is characterized by eight domains, consisting of dsRNA-binding domains (dsRBD) I and II at the N-terminus, a core helicase domain in the center and a repeated arginine and glycine-glycine-rich region at the C-terminus (Figure 1) [14]. DHX9 is an abundant, nuclear protein with a variety of putative roles including maintenance of genomic stability, DNA replication, transcription and translation [15]. It shuttles between the nucleus and cytoplasm and is capable of binding both ssDNA and ssRNA, as well as unwinding double-stranded (ds) nucleic acids and complex polynucleotide structures. While it can act on multiple nucleotide structures, it appears to have a preference for RNA displacement loops (R-loops), and both RNA and DNA G-quadruplexes [16].

### DHX9 & maintenance of genomic stability

**Figure 2.**
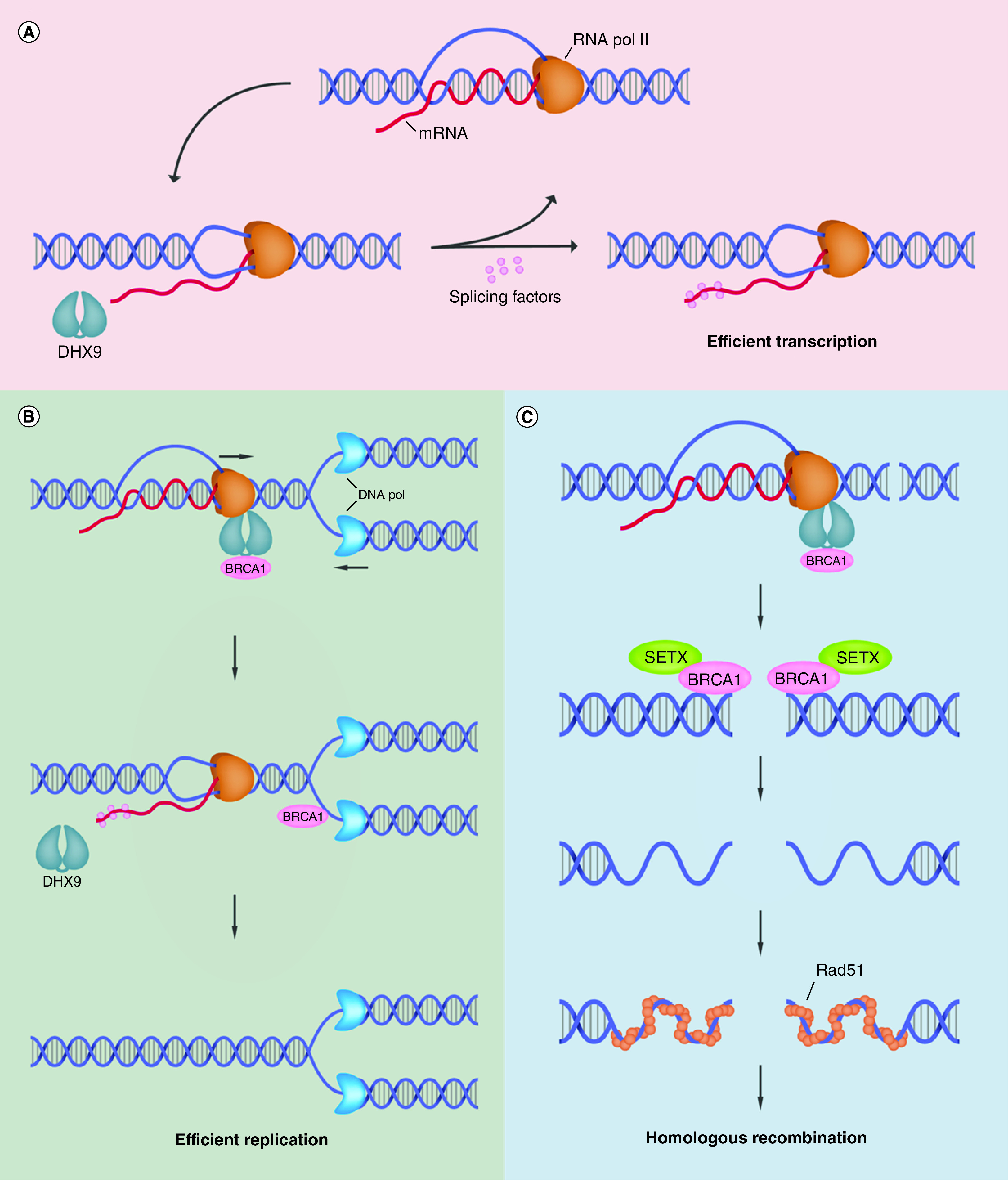
DExH-Box helicase 9-mediated R-loop resolution in the maintenance of genomic stability, transcription and DNA replication. **(A)** DHX9 associates with RNA Pol II and functions in the resolution of R-loops by unwinding nascent RNA. The unwound mRNA is then bound by splicing factors to prevent strand invasion and thus transcription continues efficiently. In the absence of splicing factors, the unwound mRNA can invade duplex DNA to re-form an RNA/DNA hybrid and generate an R-loop. **(B)** DHX9-BRCA1 interaction recruits BRCA1 to sites of transcription/replication conflicts. Upon collision of transcription and replication machinery, DHX9 functions in R-loop resolution while BRCA1 functions in protection and repair of stalled replication forks and associated DNA damage. Upon removal of R-loops, RNA Pol II can dissociate allowing efficient DNA replication. **(C)** DHX9 recruits BRCA1 to sites of dsDNA breaks. Formation of R-loops near double-stranded break sites recruits BRCA1 where it interacts with SETX in order to mediate end resection and initiate homologous recombination, resulting in repaired DNA and maintenance of genomic stability. DHX9: DExH-Box helicase 9; DNA Pol: DNA polymerase; mRNA: Messenger RNA; RNA Pol II: RNA polymerase II; SETX: Senataxin.

Genomic integrity is maintained through the DNA damage response, whereby cells undergo cell cycle arrest to initiate repair pathways or, if irreparable, induce cell death [17]. DHX9 mediates genomic stability via resolution of R-loop-associated DNA damage [3]. R-loops form during transcription when nascent RNA leaves RNA Polymerase II (RNA Pol II) and forms an RNA/DNA hybrid with the template DNA strand, subsequently displacing the nontemplate DNA strand [18,19]. While R-loops facilitate regulation of gene expression, their presence can contribute to genomic instability in several ways. For example, they can cause transcription/replication conflicts leading to stalled replication forks and DNA breaks, and their presence also leaves the displaced nontemplate strand susceptible to DNA damage [20,21]. DHX9 plays a fundamental role in maintaining genomic stability by interacting with RNA/DNA hybrids, resolving these structures and facilitating R-loop suppression [3,16,22]. Interestingly, however, a recent study revealed a conflicting ability of DHX9 to promote R-loop formation in cells with defective splicing machinery. In the absence of splicing factors to stabilize unwound nascent RNA, DHX9 activity promotes development of an RNA/DNA hybrid and subsequent generation of an R-loop (as shown in Figure 2A), thus highlighting the cellular-context-dependent mechanisms of DHX9 in regulation of genomic stability [23].

An important point to note is that DHX9’s ability to interact with DNA repair proteins allows the helicase to mediate their recruitment to R-loops in order to promote repair of R-loop-associated DNA damage. For example, DHX9 links BRCA1 to the RNA Pol II complex [24], which can explain the recruitment of BRCA1 to R-loops where it has been proposed to interact with senataxin to promote DNA repair and R-loop suppression [25,26]. Furthermore, BRCA1 is associated with stalled replication forks whereby it functions in fork protection, R-loop resolution at transcription/replication conflicts and DNA repair (Figure 2B) [27]. Interestingly, R-loops have also been shown to facilitate DNA damage repair following their formation near double-stranded breaks (DSBs) [28]. Accumulation of R-loops at these sites is essential for recruitment of BRCA1 to mediate homologous recombination (HR) [29]. Upon recognition of DNA damage, DHX9 forms a complex with BRCA1 in order to facilitate translocation to sites of DSBs. Recruitment of this complex promotes DNA end resection and subsequent RPA binding required for HR progression and DSB repair (Figure 2C) [30]. Conceptually, it is likely that DHX9-mediated recruitment of BRCA1 to RNA Pol II at transcription pause sites or DSBs functions as a complex to prevent/resolve R-loops, alleviate replication stress and mediate DNA damage repair [25,30]. Crucially, a fine balance is required between R-loop formation and resolution in order to regulate gene transcription without threatening genomic stability, and DHX9 plays a fundamental role in maintaining this balance [3,31]. Taken together, these studies highlight the critical role of DHX9 in the maintenance of genomic stability.

### DNA replication

The aforementioned role of DXH9 in the resolution of transcription/replication conflicts prevents replication stress by allowing the replication machinery to function efficiently (Figure 2B) and this highlights a role for DHX9 in regulation of DNA replication [30]. Consistent with this notion, DHX9 binds to, and enhances the activity of, an enzyme called WRN helicase that is involved in both DNA replication and the maintenance of genomic integrity [32,33]. It is suggested that the DHX9-WRN complex functions in DNA replication and/or repair to resolve structures that could impede DNA replication machinery [7]. While the exact function of DHX9 in DNA replication is unclear, DHX9 localizes to origins of replication and its knockdown hinders DNA replication, subsequently inducing p53-dependent senescence in human diploid fibroblasts. Such data frank the importance of DHX9 in this process [34].

### Transcription & translation

As noted above, R-loops modulate the regulation of gene expression and their coformation with G quadruplexes (G4s) further stabilizes structures, which can impair transcription elongation [16,35]. While DHX9 is capable of unwinding many types of nucleotide structures, it shows preference for R-loops and DNA G4s, resolving these structures to promote unhindered transcription [16]. However, it is important to note that DHX9’s conflicting ability to promote R-loop formation in the absence of splicing factors [23] exposes the dual abilities of this helicase in regulating transcription in a cell-type-specific manner (Figure 2A).

DHX9 can also influence transcriptional activation via binding to the promoter regions of numerous genes, including *p16^INK4a^* [36] and *MDR1* [37]. Likewise, DHX9 forms complexes with nuclear EGFR to mediate binding to target gene promoters following EGF-stimulated EGFR nuclear translocation [38]. While DHX9 bridging of BRCA1 to RNA Pol II [24] has been discussed above, BRCA1 is thought to function as both a transcriptional coactivator and repressor, thereby also implicating this complex in transcriptional regulation [39]. Similarly, DHX9 acts as a scaffold to bridge the transcriptional coactivator CBP (CREB-binding protein) to RNA Pol II, stimulating CREB-dependent transcriptional activation of target genes [40]. This interaction with CBP was also shown to facilitate recruitment of CBP to the p65 subunit of NF-κB following DHX9-p65 binding, stimulating NF-κB-mediated transcriptional activation [41]. However, a recent study revealed that p65 is recruited to preloaded CBP/p300 on p65-target gene promoters [42], leading to speculation that DHX9 binds p65 to facilitate recruitment to CBP at target promoter sites, rather than recruiting CBP to p65.

A major function of DHX9 is processing of secondary structures on mRNA, and an inability to resolve these structures can lead to hindered translation initiation [43]. One example is the formation of RNA G4s (rG4s) at the 5′ untranslated region (5′ UTR) of mRNA, which can cause stalling of the preinitiation complex and repress translation [44]. DHX9 regulates translational efficiency via binding and unwinding rG4 structures to stimulate the initiation of translation [44,45]. Conversely, DHX9 also participates in post-transcriptional repression via a role in RNA interference. DHX9 can promote the assembly of the RNA-induced silencing complex (RISC) to drive gene silencing [46,47]. Similarly, DHX9’s interaction with BRCA1 promotes maturation of primary transcripts of miRNA (pri-miRNA), thus participating in miRNA processing, from which miRNAs mediate translational suppression and mRNA cleavage [12,48].

### Innate immunity

The enigmatic nature of DHX9 also extends to the field of innate immunity where the helicase has conflicting roles in both the defense against viral infections and contribution to viral replication [49]. As previously stated, DHX9 mediates NF-κB-dependent gene transcription [41]. In response to DNA virus infection, DHX9 induces NF-κB-mediated transcription of antiviral cytokines in fibroblasts and epithelial cells, thus enhancing the antiviral immune response of permissive cells against DNA viruses [50]. In addition to this, DHX9 has been identified as a cytosolic viral DNA sensor in plasmacytoid dendritic cells, dependent on interaction with MyD88 [51], as well as a viral dsRNA sensor in myeloid dendritic cells [52], actions that are required to trigger NF-κB activation and subsequent induction of the antiviral cytokine response. In apparent opposition to these protective actions, DHX9 also facilitates the replication of numerous viruses, including hepatitis B virus (HBV) [53,54], hepatitis C virus [55], human immunodeficiency virus [56] and chikungunya virus [57]. In particular regarding HBV, it has recently been revealed that the HBV protein X promotes upregulated DHX9 expression, while DHX9 facilitates HBV DNA replication dependent on an interaction with Nup98 to stimulate DHX9’s helicase activity [53]. Another recent study also revealed that DHX9 promotes HBV DNA replication via inhibiting the apolipoprotein B mRNA-editing enzyme catalytic polypeptide-like 3B (A3B), which usually functions to impede HBV DNA replication [54].

## DHX9 in cancer

The hallmarks of cancer refer to biological traits acquired by cells during the development of cancer, which include enhanced proliferative signaling, evasion of growth suppression, replicative immortality, resistance to cell death, and promotion of angiogenesis, invasion and metastasis [58]. DHX9 plays a fundamental role in mediating genomic stability as well as regulating cellular processes preceding the development of numerous cancer hallmarks [16,22]. Aberrant DHX9 activity leading to genomic instability and dysregulation of molecular events can have profound pathological consequences including malignant development, and it is clear that overexpression of DHX9 is a characteristic of many cancer types [5,6,59]. From this point onward, this review will focus on the implications of DHX9 in malignant development and discuss the potential exploitation of DHX9 as an antineoplastic therapeutic target.

### DHX9 in oncogene & tumor suppressor regulation

An accumulation of genetic and epigenetic changes drives malignant development and this manifests via dysregulation of oncogenes and tumor suppressor genes in most cancers. Proto-oncogenes stimulate processes such as growth, proliferation and survival, while tumor suppressor genes promote DNA repair, cell cycle arrest, growth suppression and cell death pathways [60]. Aberrant activation of proto-oncogenes into oncogenes, and inactivation of tumor suppressor genes, drives oncogenic signaling pathways while diminishing tumor suppressive mechanisms, thus conferring a selective advantage to the tumor [61]. DHX9 activity has been identified as a factor in the transcription of both oncogenes and tumor suppressor genes in human cancers. Additionally, R-loop perturbations that occur in most cancers induce aberrant transcription of oncogenes and tumor suppressor genes, suggesting that DHX9’s function in R-loop resolution may be defective in cancer cells [62]. Furthermore, DHX9 transcriptionally regulates both oncogenes and tumor suppressor genes, or modulates the activity of their encoded proteins (see below), however, whether DHX9 functions as an oncoprotein or tumor suppressor is a current topic of debate.

#### DHX9 in sustained proliferative signaling

Sustained proliferation of cancer cells can be achieved through the upregulation of a variety of proliferative signaling pathways [58]. While there has been little work characterizing DHX9’s role in proliferative signaling, the helicase’s ability to regulate transcription of oncogenes and tumor suppressor genes could promote the activation of proliferative signaling transduction routes. A recent multiomics approach identified notable activation and connection between DHX9, EED and AURKA genomic, proteomic and metabolomic networks in breast tumors, which correlated with activation of proliferative signaling pathways, such as RAS, PI3K and Rb/E2F [63]. Furthermore, DHX9 lies downstream of the transcription factor SOX4, which suggests that DHX9 may be a SOX4 transcriptional target [64]. *SOX4* has been proposed as an oncogene because of its frequent overexpression in cancers, including prostate and breast cancer. SOX4 also has involvement in proliferative signaling pathways such as Wnt and PI3K [65–67]. In PCa, SOX4 forms a nuclear complex with plankoglobin following induction of Wnt signaling. This complex affects SOX4-DHX9 binding causing DHX9 suppression, highlighting SOX4’s role in modulating DHX9 expression in PCa progression and the SOX4-DHX9 complex’s role in Wnt signaling [68].

#### DHX9 in evasion of growth suppressors & replicative immortality

**Figure 3.**
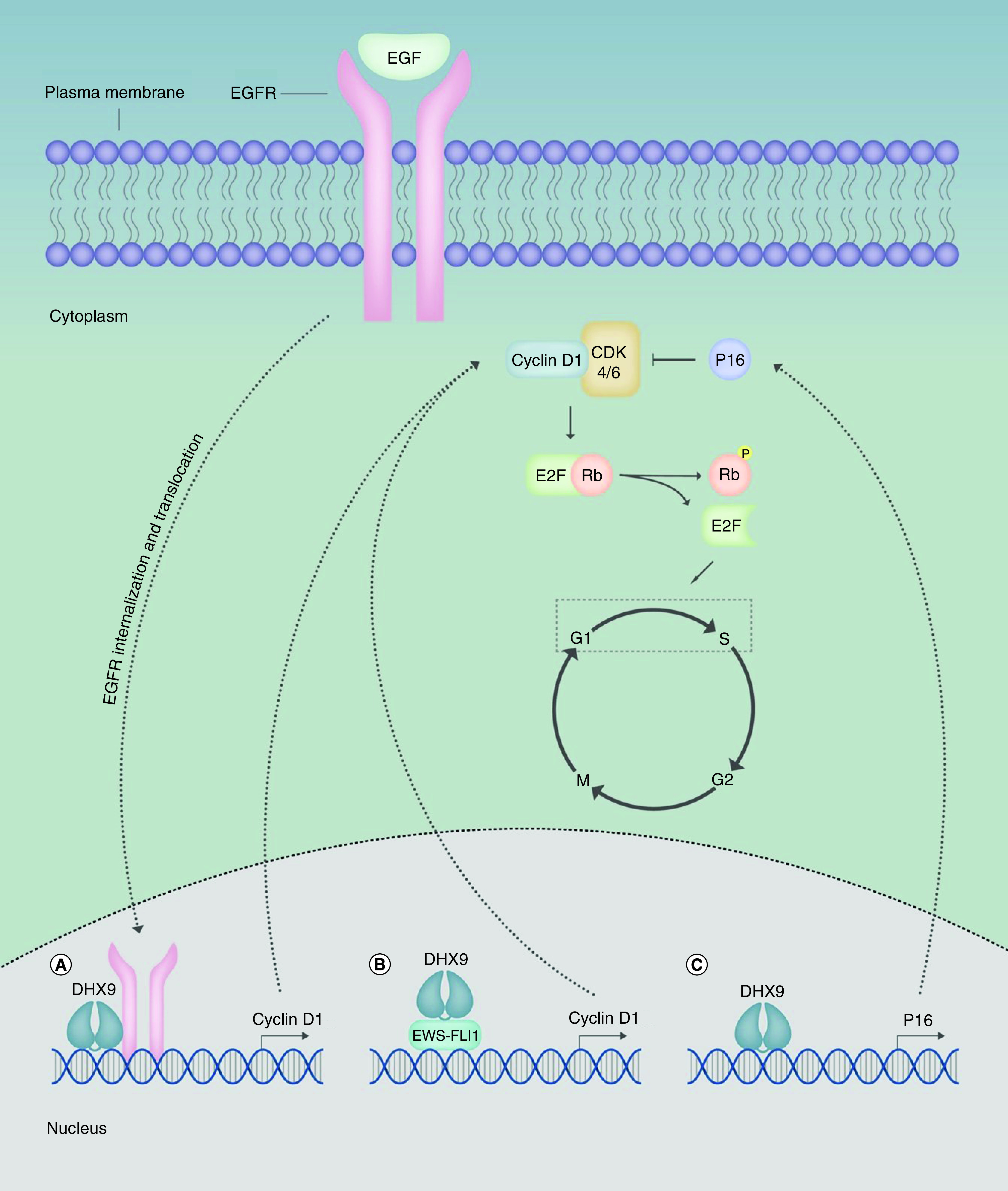
DExH-Box helicase 9 participates in the transcription of both cyclin D1 and p16^INK4A^, exhibiting both oncogenic and tumor suppressive functions. **(A)** Following EGF-stimulated nuclear translocation of *EGFR*, DHX9 facilitates EGFR-mediated cyclin D1 transcription. **(B)** DHX9 interacts with EWS-FLI1 in Ewing sarcoma cells, promoting EWS-FLI1-mediated cyclin D1 transcription. **(C)** DHX9 binds to the p16^INK4a^ promoter, functioning as a transcriptional coactivator to induce *p16* transcriptional. Cyclin D1 binds CDK4/6 to induce dissociation of Rb and E2F, stimulating G1/S transition to promote replicative immortality. P16^INK4A^ functions as a tumor suppressor by inhibiting the cyclin D1-CDK4/6 complex, thus allowing Rb to inhibit E2F-mediated transcription of genes promoting G1/S transition. In cancer cells, the interaction between DHX9 and p16^INK4A^ is often suppressed, thus diminishing its tumor suppressive abilities. CDK4/6: Cyclin-dependent kinase-4/-6; DHX9: DExH-Box helicase 9; EGFR: EGF receptor; *p16*: p16INK4a; Rb: Retinoblastoma protein.

Evasion of growth suppression and dysregulated cell cycle control not only leads to replicative immortality but can also contribute to cell proliferation [58]. Overexpression of the *EGFR* oncogene has been identified in many cancers that are a product of aberrant EGFR signaling [69]. As previously mentioned, DHX9 mediates EGFR binding to target gene promoters, including the *CCND1* proto-oncogene that stimulates cyclin D1 transcriptional activation (Figure 3A), as observed in human breast cancer cells [38,70]. Cyclin D1 is a cell cycle regulator that promotes G1/S transition upon interaction with cyclin-dependent kinase-4/-6 (CDK4/6). The retinoblastoma protein (Rb) functions as a growth suppressor, by binding E2F transcription factors to repress E2F-target gene transcription. The cyclin D1-CDK4/6 complex catalyzes hyperphosphorylation of Rb to inhibit its activity in order to stimulate cell cycle progression and cell proliferation [71]. Overexpression of cyclin D1 is frequently observed in cancers, including breast [72], pancreatic [73] and lung cancer [74]. It therefore appears that DHX9 may contribute to the overexpression of cyclin D1 in cancer following oncogenic EGFR upregulation, thus promoting proliferation and replicative immortality. DHX9 also forms a cocomplex with RNA Pol II and EWS–FLI1 to augment EWS–FLI1-dependent *CCND1* transcription (Figure 3B) [15]. Interestingly, however, upon inhibition of this interaction, DHX9 associates with Sam68, recruiting it to *pncCCND1_B* (a *CCND1* promoter-associated noncoding RNA), ultimately repressing *CCND1* transcription. This work highlights DHX9’s ability to form alternative complexes with either EWS-FLI1 or *pncCCND1_B*/Sam68 in modulating *CCND1* expression in Ewing sarcoma cells, exhibiting both oncogenic and tumor suppressive functions [75]. In this context, it should also be noted that DHX9 binds to the p16^INK4a^ promoter and functions as a transcriptional coactivator (Figure 3C) [36]. p16^INK4a^ is a tumor suppressor that functions to inhibit the cyclin D1-CDK4/6 complex thus, promoting cell cycle regulation, however, in cancer cells this interaction is often suppressed, leading to transcriptional silencing of p16^INK4a^ that results in uncontrolled G1/S transition and replicative immortality [36,76]. This highlights the tumor suppressive role of DHX9 in modulating p16^INK4a^ transcription on cell cycle control. In a similar vein, DHX9 can also mediate CDK6 downregulation, thereby supporting the helicase’s tumor suppressive functions that are compromised in hepatocellular carcinoma (HCC) due to the overexpressed long noncoding (lnc) RNA lnc-UCID. CDK6 binds to lnc-UCID sequestering the kinase from DHX9, preventing DHX9-mediated CDK6 downregulation and leading to dysregulated G1/S transition and uncontrolled cell proliferation [59].

DHX9 is also capable of suppressing activity of the tumor suppressor BRCA1, which functions in the DNA damage response and cell cycle arrest [77,78]. BRCA1 interacts with hypophosphorylated Rb, which is suspected to maintain Rb in its E2F-bound activated state, preventing E2F-target gene transcription and inhibiting cell proliferation [79]. As previously discussed, DHX9 interacts with BRCA1 to coordinate HR and maintain genomic integrity [30], however, overexpression of a DHX9 peptide fragment containing the region for BRCA1 interaction was shown to inhibit BRCA1 activity in breast epithelial cells and promote breast cancer progression [77]. Additionally, truncated DHX9 mutants affect the interaction between BRCA1 and RNA pol II, subsequently leading to decreased BRCA1 transcription [80]. Decreased BRCA1 transcription can result in defective cell cycle checkpoints and enhanced proliferation [78,81]. Taken together, these studies reveal that DHX9 can contribute to BRCA1 suppression and subsequent Rb dysregulation, promoting genomic instability, replicative immortality and proliferation.

#### DHX9 in evasion of cell death

p53 is a tumor suppressor that has been named ‘the guardian of the genome’ as it promotes cell cycle arrest or induction of apoptosis upon detection of DNA damage via enhanced production of proapoptotic proteins. p53 is commonly mutated or deficient in cancer, therefore allowing damaged cells to evade apoptosis, accumulate and promote cancer progression [82]. As previously noted, DHX9 is overexpressed in many cancers and inhibition of DHX9 evokes p53-mediated apoptosis in numerous human cancer cell lines, highlighting DHX9’s involvement in this pathway [83]. Interestingly, a follow-up study also identified p53-independent induction of cell death following *DHX9* inhibition in p53-deficient mouse lymphomas and colon cancer cells, highlighting the dependency of both p53-wildtype and p53-deficient tumors on DHX9 for survival [83,84]. Conversely, nuclear DHX9 is essential for KIF1B isoform β (KIF1Bβ)-mediated neuronal apoptosis. In cells with aberrant nerve growth factor signaling, the tumor suppressor KIF1Bβ mediates nuclear localization and accumulation of DHX9, which is necessary for KIF1Bβ-induced apoptosis of neuronal cells. However, *KIF1Bβ* is often deleted in neuroblastoma tumors, thus impairing nuclear DHX9 localization and subsequently allowing neuroblastoma cells to evade apoptosis [85]. This work further highlights the context-dependent manner of DHX9 in promoting either tumor suppression or tumorigenesis and adds another layer of contradiction to this enigmatic helicase.

#### DHX9 in angiogenesis, metastasis & invasion

One method by which DHX9 activity has been shown to influence cancer cell invasion is via interactions with lncRNAs leading to modulation of their activity. Lnc-cervical cancer DHX9 suppressive transcript (lnc-CCDST) binds DHX9, acts as a scaffold to enhance MDM2-DHX9 binding and thus induces DHX9 degradation. In cervical cancer (CC) cells, lnc-CCDST is downregulated via the human papillomavirus-encoded E6 and E7 oncoproteins, allowing DHX9 to accumulate and result in enhanced invasion and angiogenesis [86]. DHX9 is also implicated in the production and regulation of circRNAs, another type of noncoding RNA which can serve as miRNA sponges in order to regulate gene expression [87,88]. Recently, it has been discovered that DHX9 phosphorylation can promote upregulation of oncogenic circCCDC66, which correlates with colorectal cancer (CRC) growth, invasion and metastasis [89,90]. However, circRNAs can also possess tumor suppressive functions, for example, in the case of cSMARCA5 that inhibits growth and migration of HCC cells. DHX9 upregulation induces cSMARCA5 downregulation, suppressing its tumor suppressive functions to promote HCC progression and metastasis [91]. Overexpression of DHX9 has previously been identified in osteosarcoma cells exhibiting high metastatic ability [92], and these studies highlight the ability of DHX9 to both promote oncogenic and inhibit tumor suppressive circRNAs to promote invasion, migration and metastasis in CRC and HCC. Additionally, circ0005276 has been associated with proliferation, invasion and migration in PCa. circ0005276 regulates *XIAP* transcription via interaction with FUS, with both circ0005276 and *XIAP* overexpressed in PCa cells and tissues [93]. Interestingly, DHX9 has been shown to interact with FUS in HEK293 cells [94], which positions DHX9 to mediate FUS-circ0005276-mediated *XIAP* expression, and subsequent PCa invasion and migration.

Epithelial–mesenchymal transition (EMT) is a critical cellular process occurring during development that is characterized by a shift from epithelial to mesenchymal phenotype, however, cancer cells can hijack this event to promote transition to the more invasive mesenchymal phenotype, permitting escape from the primary tumor, invasion into the bloodstream and metastasis to distant locations [95]. Many different circRNAs have been associated with EMT in cancer cells [96], and DHX9 plays fundamental roles in modulating circRNAs during EMT. For example, DHX9 expression in bladder cancer (BC) tissue suppresses circPICALM action, which usually functions as a tumor suppressor by ‘sponging’ for miR-1265, preventing FAK phosphorylation and EMT. This is a prime example of how DHX9 overexpression can overcome tumor suppressive protection in BC and negatively influence EMT to promote BC invasion and metastasis [97]. Furthermore, as previously noted, DHX9 phosphorylation augments circCCDC66 expression in CRC [90], and circCCDC66 levels have been associated with the mesenchymal phenotype in lung cancer, which is likely regulated by FAK [98]. Therefore, DHX9’s involvement in circCCDC66 regulation, and putative cross-talk with FAK phosphorylation and EMT upon DHX9-mediated circPICALM suppression, further implicates it in cancer cells’ acquisition of a mesenchymal phenotype and enhanced invasive features. As with most functions of DHX9, there is a converse role for the helicase in lung adenocarcinoma where DHX9 functions as a tumor suppressor and inhibits EMT. *DHX9* knockdown in these cells induced STAT3 phosphorylation and subsequent proliferation, migration and invasion *in vitro* [5]. Upregulated STAT3 has been shown to promote EMT in non-small-cell lung cancer [99], therefore, it is speculated that DHX9 mediates EMT via STAT3 regulation in lung adenocarcinoma [5].

## DHX9 as a biomarker & therapeutic target

### DHX9 as a biomarker for chemotherapy resistance

While the literature franking the involvement of DHX9 in the development and progression of cancer is robust and plentiful, a newly discovered feature of DHX9 revolves around resistance to chemotherapeutic agents. The majority of chemotherapy drugs, such as platinum-derived agents, target highly proliferative cells and induce DNA damage that triggers cellular DNA damage responses and repair pathways, however, upon irreversible DNA damage cell death is programmed via apoptosis [100]. Resistance to DNA-damage inducing chemotherapeutics can occur via upregulated DNA repair responses leading to sufficient repair and oncogenic cell survival, therefore, DHX9’s aforementioned involvement in regulating these processes position the helicase as an influencer of chemotherapy [3,30,101]. It is thought that post-translational modification of DHX9 occurs in drug-resistant cells, with a higher molecular weight observed in drug-resistant than drug-sensitive leukemic cells [102]. DNA-PK-mediated DHX9 phosphorylation upregulates *MDR1* expression, and subsequently drives multidrug resistance in leukemia cells [37]. Additionally in CRC, DHX9-mediated circCCDC66 upregulation promotes resistance to oxaliplatin, while inhibiting DHX9 phosphorylation suppressed circCCDC66 expression and chemoresistance, highlighting DHX9 activity in the development of oxaliplatin-resistant CRC cells [90]. Contrary to this, a proteomics-based study identified DHX9 as a key protein downregulated in temozolomide-resistant glioblastoma, and that the spliceosome signaling pathway was affected in these cells [103]. Given that DHX9 is regulated via alternative splicing [15], dysregulation of this pathway may aberrantly affect DHX9 synthesis and promote temozolomide-resistance. This study recognized DHX9’s potential as a biomarker in glioblastoma, with low expression correlating with a less favorable prognosis. Another study identified DHX9 as a binding partner of Schlafen-11 (SLFN11), a proposed predictive biomarker of resistance to platinum-based chemotherapeutics in ovarian and lung cancers. It is speculated that diminished *SLFN11* expression compromises the DHX9-BRCA1 interaction, altering the regular DNA damage repair pathway and promoting chemoresistance [101].

### Targeting DHX9 to mediate chemosensitivity

Analysis of a cadre of RNA/DNA hybrid-binding proteins has revealed that DHX9 has one of the highest levels of drug sensitivity interactions with the US FDA-approved chemotherapeutic agents, exhibiting both positive and negative correlations, highlighting the fact that DHX9 expression can variably affect chemotherapy response [104]. p53-induced apoptosis is an essential mechanism for cancer cell death induction following chemotherapy-induced DNA damage and this is the reason why P53 has been strongly liked with chemosensitivity [105]. DHX9’s involvement in the p53 pathway has previously been discussed, with DHX9 inhibition evoking p53-mediated apoptosis in numerous cancer cell lines [83]. Similarly, silencing *DHX9* induces activation of the p53 signaling pathway in a mouse lymphoma model. In this context, the activated replicative stress response induced apoptosis and sensitized resistant lymphoma cells to ABT-737 [106]. Conceptually, DHX9 could be targeted alongside ABT-737 treatment as a feasible strategy to overcoming resistant lymphoma cells. Additionally, DHX9 expression can be tuned to improve chemosensitivity of Ewing sarcoma cells. As previously discussed, DHX9 binds EWS-FLI1 in Ewing sarcoma, enhancing EWS-FLI1-dependent transcription [107]. Inclusion of poison-exon 6A in *DHX9* would usually target it to nonsense-mediated mRNA decay, however, in Ewing sarcoma *DHX9* expression is upregulated following repression of this exon and correlates with a poor prognosis [15]. A different study revealed that SRSF3 and hnRNPM can bind to *DHX9* exon 6A to suppress its inclusion and promote *DHX9* expression. Downregulation of these proteins inhibits *DHX9* expression, suppresses proliferation and sensitizes Ewing sarcoma cells to doxorubicin treatment, revealing the importance of DHX9 alternative splicing in conferring chemosensitivity or chemoresistance [108].

### DHX9 as an antineoplastic target for inhibition

One of the main pitfalls of chemotherapy is lack of specificity due to the general targeting of highly proliferative cells as opposed to only bona fide cancer cells, which can result in highly toxic cellular conditions that inadvertently damage healthy tissue [109]. Targeted inhibition of proteins implicated in tumorigenesis is a promising anticancer therapy because of the heightened selectivity of the approach. Given that DHX9 is overexpressed in many cancers and contributes to the development of numerous hallmarks of cancer (as previously discussed), the helicase should represent an attractive antineoplastic target. While Lee *et al.* revealed that *DHX9* depletion in mice induced embryonic lethality, thus proving essential for embryonic development [110], several studies exploring inhibition of DHX9 in cancer cells have discovered that it is not only effective but also safe in adult mice. For example, shRNA-mediated *DHX9* knockdown was lethal to a number of cancer cell lines and lymphoma cells of a mouse model while imposing no detrimental effects on healthy tissue *in vivo*. Additionally, adult mice seemed to tolerate long-term genetic *DHX9* suppression. Of particular importance in this regard is the fact that *in vivo* *DHX9* knockdown did not negatively affect other highly proliferative cells, ensuring high specificity while exhibiting low toxicity to healthy tissue [83]. A follow-up study then highlighted the potential of targeting DHX9 in p53-deficient tumors, whereby *DHX9* suppression in p53-null tumors also induced cell death or cell cycle arrest [84]. Given that p53 is commonly inactivated in cancer cells [111], this validates the approach of targeting DHX9, which could be broadly applied to many cancers. With this in mind, an aptamer-targeting DHX9 has been identified for use in CRC tumor cells *in vivo*. The aptamer binds DHX9 with high affinity and specificity allowing preferential action in cancer cells over normal tissue due to DHX9 overexpression in CRC cells. Further specificity is achieved by localizing the aptamer to the nucleus allowing targeted delivery and this approach did not negatively affect normal cells. Given that this work was performed in human xenografts, it is highly likely that such a method could translate to humans, further establishing DHX9 as an effective and safe molecular target [6]. Another study investigating the overexpression of DHX9 in lung cancer highlighted the potential use of enoxacin, a fluoroquinolone, for targeting DHX9. It is speculated that enoxacin enhances RNA interference dependent on DHX9 expression in non-small-cell lung cancer cells, inhibiting their proliferation [4]. Further research into combining enoxacin with other chemotherapeutic agents may be a future avenue to explore in cancer treatment.

### DHX9 in oncolytic viral therapy

Cancer immunotherapy can offer more selective and tailored treatment than chemotherapy by hijacking the immune system to recognize and destroy cancer cells [109]. JP Allison and T Honjo won the 2018 Nobel Prize for their discovery of immune checkpoint blockade, which has redefined treatment options for many cancers, including melanoma, through the use of monoclonal antibodies targeting CTLA-4 and PD-1/PD-L1 [112]. Oncolytic viruses (OVs) are a novel type of immuno-oncotherapy, which can selectively replicate in tumor cells with high specificity to elicit an effective antitumor immune response [113]. Clinical trials combining immune checkpoint blockade with OVs have achieved markedly higher efficacy than monotherapy and appear tolerable without unexpected additional adverse events in patients with advanced melanoma [114,115], therefore, combined therapy has the potential to minimize off-target toxicity, as well as being applicable to a larger range of cancers.

DHX9’s aforementioned roles in innate immunity with respect to antiviral immune responses and viral replication places the helicase in a pivotal position to hone cancer cells’ responses to oncolytic viral therapies. Myxoma virus (MYXV) is a potential candidate for oncolytic viral therapy because of its ability to infect human cancer cells both efficiently and safely [116,117]. Rahman *et al.* identified that DHX9, along with several other DExD/H-box helicases, provokes antiviral responses by attenuating MYXV viral replication in human cancer cells. siRNA-mediated *DHX9* knockdown enhanced MYXV replication in cancer cells, showing that DHX9’s regulatory role in the antiviral immune response is highly relevant in this context [118]. However, as with all aspects of DHX9 functionality, there are two sides to every coin. DHX9 can elicit both pro- and anti-viral effects. For example, an earlier study revealed proviral effects of DHX9 following its interaction with MYXV-encoded protein M029 in several human cancer cell lines, an action which promoted viral replication. M029 also binds PKR, leading to inhibition of antiviral signaling [119]. Interestingly, DHX9 is a substrate of PKR-mediated phosphorylation [120], therefore, DHX9 can also promote the MYXV viral life cycle upon repression of PKR, highlighting the dual functions of the enzyme in MYXV regulation. DHX9 also facilitates HBV replication via Nup98 regulation, and it was shown that cells infected with HBV contained upregulated DHX9 expression, with levels correlating to viral load [53]. It was then later revealed that DHX9 interacts with A3B in order to negatively regulate anti-HBV function, therefore contributing to HBV viral DNA replication [54]. Overall, given DHX9’s regulatory role in the life cycle of many viruses used as OVs, DHX9 could be targeted to enhance cancer cells’ response to OVs, thereby widening the applications of targeting DHX9 in cancer therapy to modulate cancer immunotherapy.

## Conclusion

It is clear from the literature cited above that DHX9 has well-defined roles as an oncogene and a tumor suppressor in different tissues and cancer cell types. The versatility of the helicase is a product of its multiple functions in resolving nucleotide structures and also of the numerous protein–protein interactions it makes with binding partners that transduce vital signals for fundamental cellular processes such as gene expression and DNA replication. DHX9’s multitude of functions in the development of numerous hallmarks of cancer highlight a pivotal role in malignancy and potential as both a biomarker and selective target for cancer therapy.

## Future perspective

There is growing evidence to suggest that DHX9 would make an effective therapeutic target and possibly a diagnostic gene for some cancers, however, careful thought is needed to hone approaches that selectively focus on the oncogenic potential of the helicase rather than the plethora of vital ‘housekeeping’ functions it undertakes to maintain the stability of the human genome. The proposal of DHX9 as a potential target for enhancing oncolytic viral therapy is an avenue to be further explored and given the recent advances in cancer immunotherapy, it can be assumed that this field will continue to evolve rapidly. Ultimately, targeting DHX9 for inhibition appears to offer a safe and selective approach for cancer treatment, and combined with current cancer therapies could lead to more effective and specific treatments applicable to a broad range of cancers, however, clinical trials are lacking in this area, therefore further research is required to fully ascertain its full potential.

**Executive summary**

- The hallmarks of cancer are features of cancer cells that promote/support the development and progression of cancer.
- DExH-Box helicase 9 (DHX9)’s enigmatic functions include maintenance of genomic stability, DNA replication and gene expression. Dysregulated DHX9 activity leads to a lack of coordination of these important cellular processes, which in turn can lead to the development of numerous cancer hallmarks and malignancy.
- DHX9 can function as either an oncogene or tumor suppressor dependent on its interaction with downstream partners and activation state of inter-connected signaling pathways.
- The involvement of DHX9 in malignancy positions it as an attractive biomarker and target for cancer therapy.
